# Recovery of 1887 metagenome-assembled genomes from the South China Sea

**DOI:** 10.1038/s41597-024-03050-4

**Published:** 2024-02-13

**Authors:** Shuaishuai Xu, Hailong Huang, Songze Chen, Zain Ul Arifeen Muhammad, Wenya Wei, Wei Xie, Haibo Jiang, Shengwei Hou

**Affiliations:** 1 Department of Ocean Science & Engineering, Southern University of Science and Technology, Shenzhen, 518055 China; 2 College of Life Science and Technology, Jinan University, Guangzhou, 510632 China; 3 School of Marine Sciences, Ningbo University, Ningbo, 315211 China; 4 Shenzhen Ecological and Environmental Monitoring Center of Guangdong Province, Shenzhen, 518049 China; 5 School of Marine Sciences, Sun Yat-sen University, Guangzhou, 510632 China; 6 Southern Marine Science and Engineering Guangdong Laboratory (Zhuhai), Zhuhai, 519000 China

**Keywords:** Marine biology, Microbial biooceanography, Microbial ecology

## Abstract

The South China Sea (SCS) is a marginal sea characterized by strong land-sea biogeochemical interactions. SCS has a distinctive landscape with a multitude of seamounts in its basin. Seamounts create “seamount effects” that influence the diversity and distribution of planktonic microorganisms in the surrounding oligotrophic waters. Although the vertical distribution and community structure of marine microorganisms have been explored in certain regions of the global ocean, there is a lack of comprehensive microbial genomic surveys for uncultured microorganisms in SCS, particularly in the seamount regions. Here, we employed a metagenomic approach to study the uncultured microbial communities sampled from the Xianbei seamount region to the North Coast waters of SCS. A total of 1887 non-redundant prokaryotic metagenome-assembled genomes (MAGs) were reconstructed, of which, 153 MAGs were classified as high-quality MAGs based on the MIMAG standards. The community structure and genomic information provided by this dataset could be used to analyze microbial distribution and metabolism in the SCS.

## Background & Summary

The South China Sea (SCS) is the largest marginal sea in the western Pacific Ocean. It is characterized by a tropical and subtropical climate^1^ with complex physical and chemical gradients over spatial scales^2,3^. The SCS encompasses a multitude of underwater seamounts rising from the seafloor^4,5^, which are unique topographic features that could alter the local hydrodynamics of the surrounding waters^6–8^. These seamounts cause “seamount effects” in the oligotrophic oceans, leading to intensified vertical movements and rapid exchanges of shallow and deep waters^7–10^. These vertical movements, both upwelling and downwelling, have a fundamental influence on the primary production and phytoplankton diversity^8–12^. The differential distribution patterns of diverse marine phytoplankton may further affect the assemblage of heterotrophic microbial communities as a result of substrate-constrained partition and succession^13^. For instance, it was found that the vertically distributed phytoplankton had a significant influence on the bacterioplankton community structure at different water layers surrounding seamounts in the western Pacific Ocean^8^.

The Xianbei seamount is a shallow underwater mountain situated in the central basin of the SCS, with its summit lying approximately 208 meters below the sea surface^12,14^. The deep seawater in the SCS is mainly transported from the western Pacific Ocean through the Luzon Strait^4,5^. This transportation process results in a rapid basin-scale cyclonic circulation pattern and creates deep upwelling events in the seamount regions along the way^4,5^. Mount Xianbei is one of the largest seamounts close to the euphotic zone, making it a natural laboratory for studying seamount effects on microbial diversity and distribution. In addition, how the microbial communities in seamount regions differ from those in the continental shelf or coastal waters has not been fully understood.

**Fig. 1:**
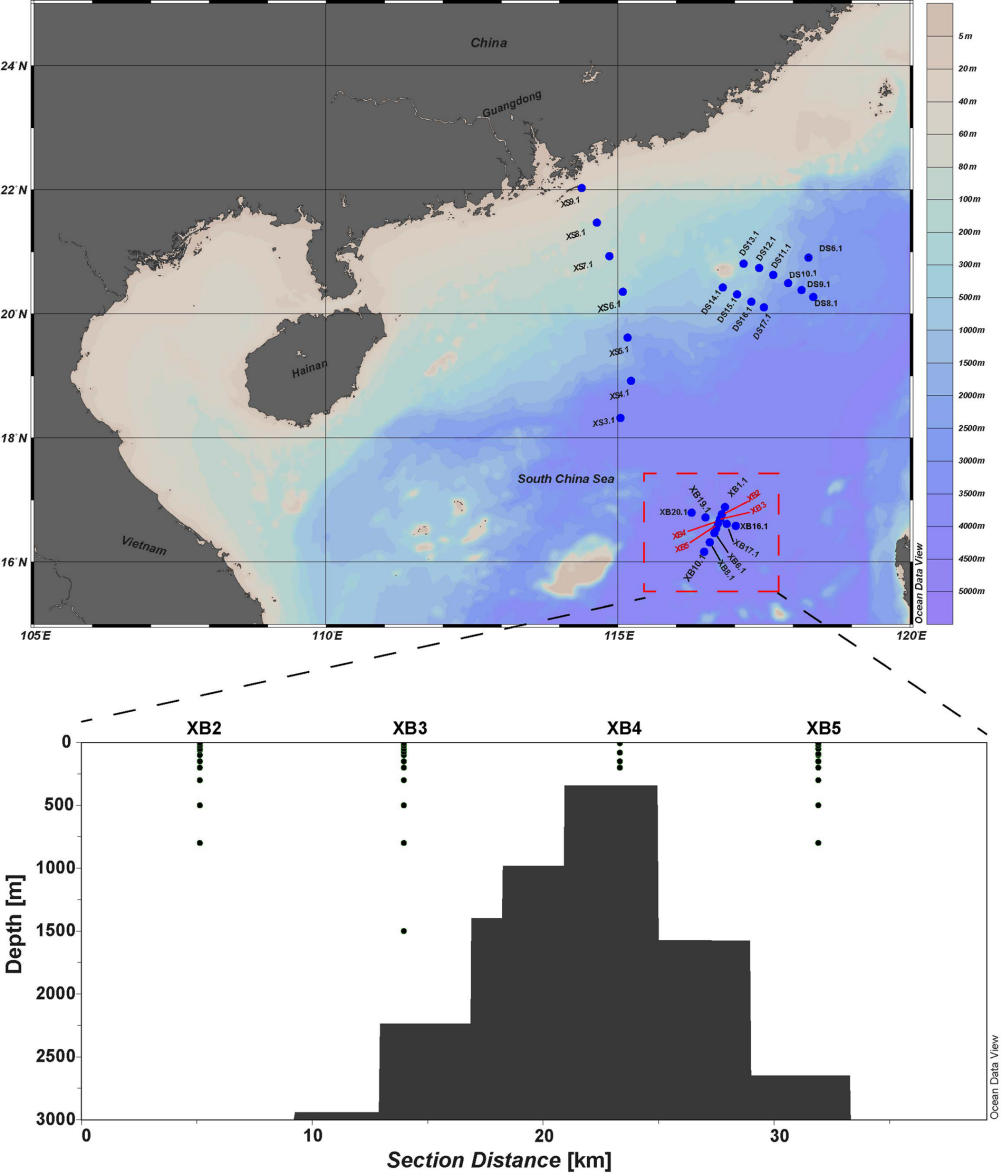
Sampling sites in the Xianbei, Xisha and Dongsha areas in SCS. The red dots shown in the upper subplot were stations with samples taken from multiple water depths as shown in the lower panel. XB: Xianbei, XS: Xisha, and DS: Dongsha.

**Fig. 2:**
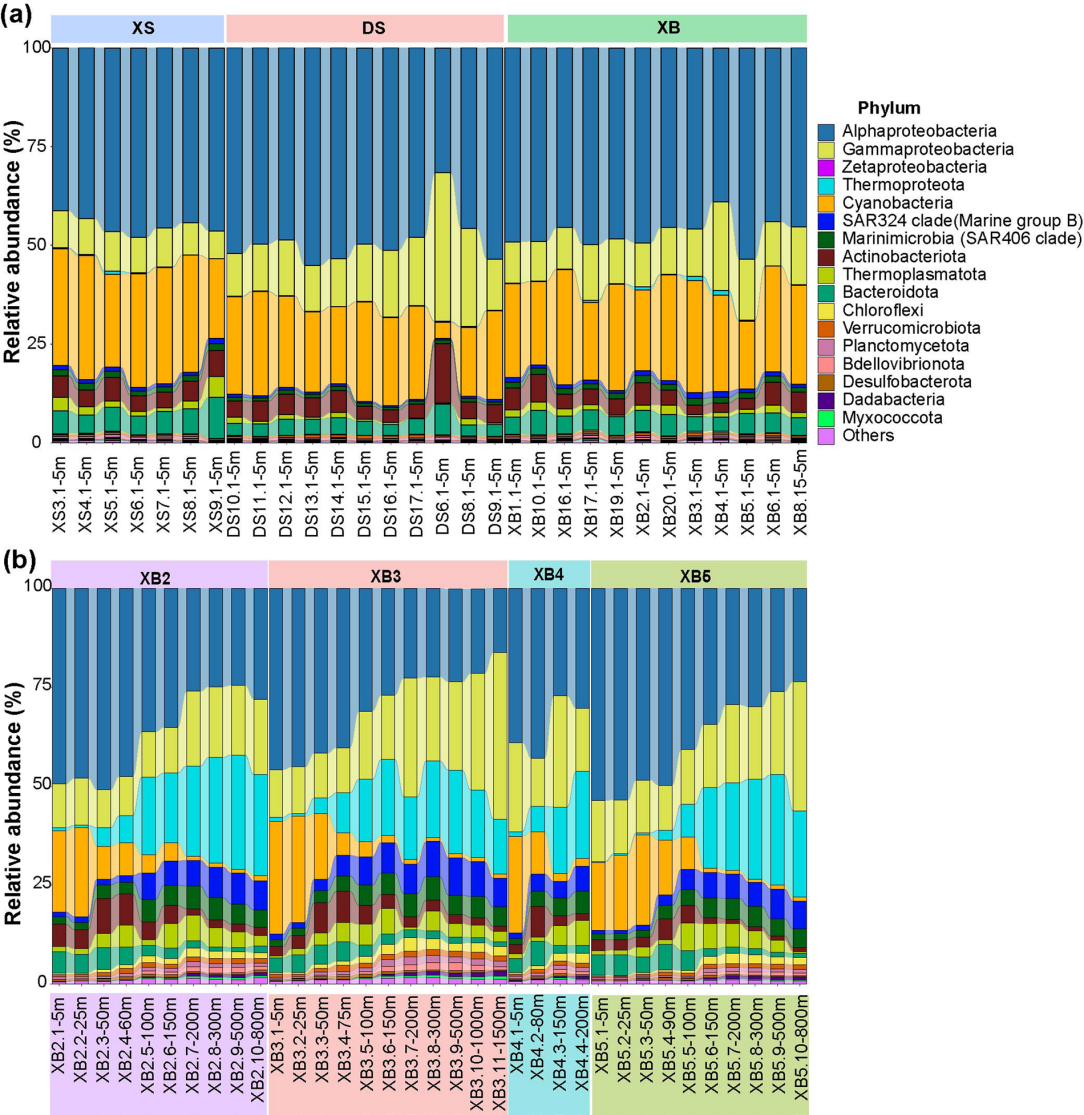
Relative abundances of microbial communities in the Xianbei, Xisha and Dongsha areas of SCS. The relative abundances of different taxa were assessed based on 16S rRNA gene amplicon sequencing across different areas (**a**) or across depths in the Xianbei seamount region (**b**). Detailed relative abundance and 16S rRNA gene taxonomy information can be found in Table S2.

In this study, we collected 61 seawater samples from the Xianbei seamount region (XB, n = 43), as well as Dongsha (DS, n = 11) and Xisha (XS, n = 7) areas to survey the microbial diversity and metabolic potentials in SCS (Fig. 1). Sample metadata, sequencing strategy and environmental factors can be found in Table S1. The 16S rRNA gene amplicon sequencing data revealed that Alphaproteobacteria and Gammaproteobacteria were the most abundant bacterial groups in all surface (5 m) samples. The cumulative relative abundance of Alphaproteobacteria Amplicon Sequence Variants (ASVs) ranged from 31.66% to 55.08%, while for Gammaproteobacteria, the cumulative proportions of ASVs were in the range of 6.98% to 37.62%. As expected, cyanobacteria were found to be prevalent in samples of the top 150 m in depth (Fig. 2a,b). In the Xianbei seamount region, as the depth increased, the cumulative relative abundance of Alphaproteobacteria or Cyanobacteria ASVs showed a decreasing trend, whereas for other taxonomic groups, such as Gammaproteobacteria, Thermoproteota, SAR324 clade, and Marinimicrobia (SAR406 clade), an increasing trend with depth was observed (Fig. 2b,Table S2).

**Fig. 3:**
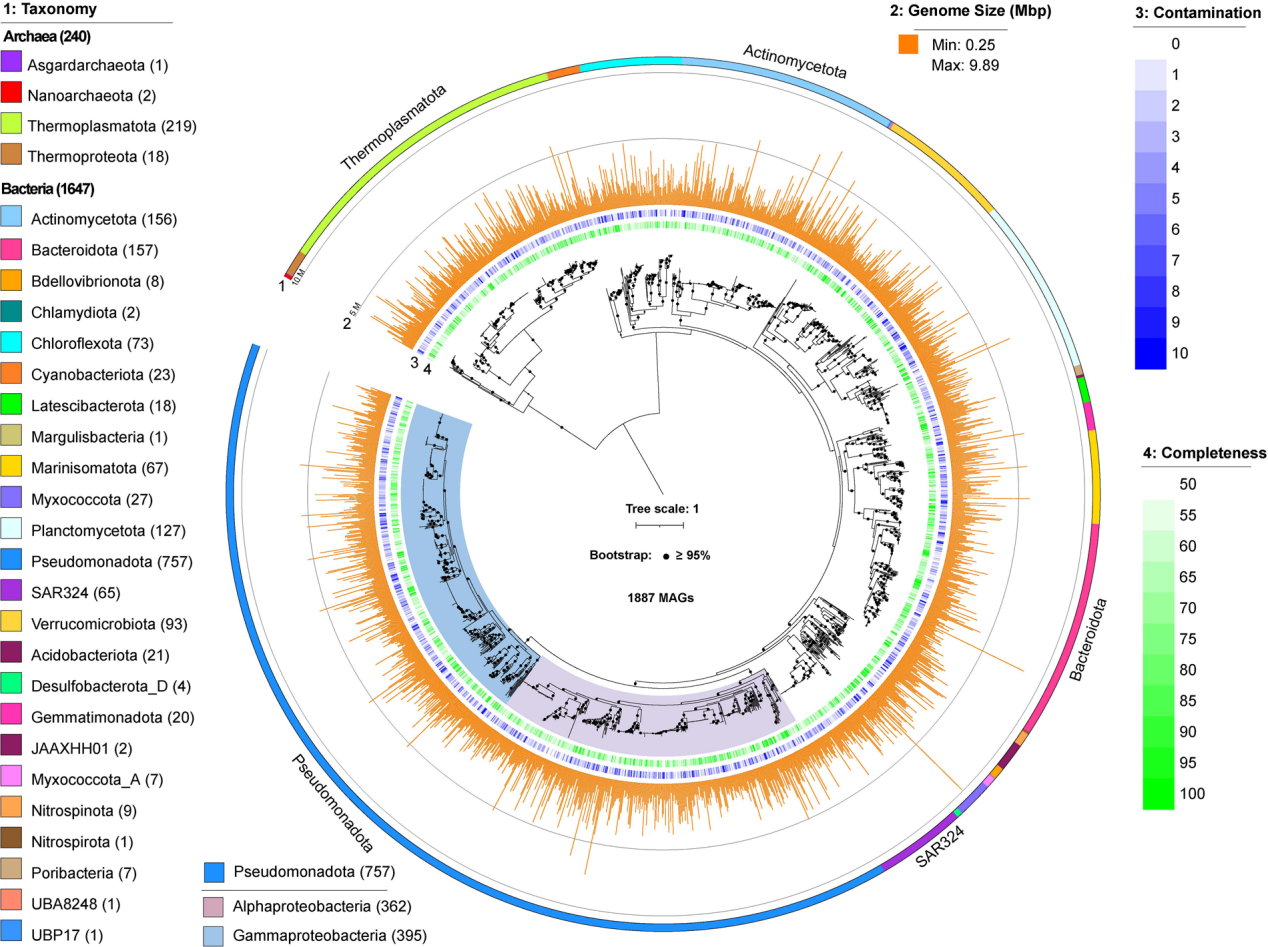
The phylogenomic tree of 1887 MAGs recovered from this study. The maximum likelihood tree was reconstructed based on the concatenated alignment of 41 single-copy marker genes. Numbers in the parenthesis after each phylum name indicate the number of recovered MAGs from this phylum. Branches with bootstrap values >0.95 were highlighted with black dots. Detailed MAG taxonomy assignment, associated with completeness and contamination information can be found in Table S3.

Upon metagenomic sequencing and binning, a total of 1887 dereplicated Metagenome Assembled Genomes (MAGs) were reconstructed with completeness ≥50% and contamination <10%. Of them, 1260, 325, and 302 representative MAGs originated from XB, DS, and XS metagenomes, respectively (Table S3a). Notably, 153 of them (8.1%) were classified as high-quality MAGs based on the MIMAG (Minimum Information about a Metagenome-Assembled Genome) standards^15^. These MAGs were taxonomically assigned to 4 archaeal and 24 bacterial phyla based on the Genome Taxonomy Database (GTDB)^16^, with a total of 240 archaeal and 1647 bacterial MAGs. Archaeal MAGs were affiliated with Thermoplasmatota (219), Thermoproteota (18), Nanoarchaeota (2), and Asgardarchaeota (1) phyla (Fig. 3, Table S3b). Bacterial MAGs were mainly from Pseudomonadota (757), Bacteroidota (157), Actinomycetota (156), Planctomycetota (127), Verrucomicrobiota (93), Chloroflexota (73), Marinisomatota (67) and SAR324 (65) phyla. Within the Pseudomonadota phylum, MAGs were assigned to either Alphaproteobacteria (362) or Gammaproteobacteria (395) class. Comparative analysis of the MAGs recovered here with those recovered from diverse SCS habitats^17–19^, OceanDNA^20^ and Tara Oceans^21^, revealed that 19.34% of the MAGs (366 MAGs) recovered in this study were not present in any of these datasets at a 95% average nucleotide identity (ANI) threshold (Table S3c).

Genes were called at the contig level and deduplicated in order to generate a non-redundant reference gene catalog, as a supplement to the MAG-based analysis. In total, 10,551,413 unique genes were predicted, and their functions were annotated with KEGG Orthology (KO) groups.

## Materials and Methods

### Sample collection and environmental variable characterization

Seawater samples were collected from the South China Sea (16°32′–16°46′ N, 116°41′–116°47′ E) between August and September, 2021. Details of sampling sites and depths can be found in Fig. 1 and Table S1. Following the methodology of a previous study on harmful algal species^12^, seawater samples were collected at a depth of 5 meters from XS3.1 to XS9.1, DS6.1 to DS17.1, and XB1.1 to XB20.1. Additionally, in the XB2, XB3, XB4, and XB5 regions, seawater samples were collected across multiple depths including 5, 25, 100, 150, 200, 300, 500, 800, 1000, and 1500 meters. 2 L seawater samples were collected from each sampling site using size-fractionated filtration to remove mesozooplankton and suspended particles, and microbial cells within the size range of 0.2–200 μm were collected on polycarbonate membrane filters (Millipore, USA). Filters were then snap-frozen in liquid nitrogen and stored at −80 °C until DNA extraction. Temperature (°C), and Density (Kg/m³^3^) were measured using a SeaBird CTD system (Ocean Test Equipment, Florida, USA) on board.

### DNA extraction, amplicon and metagenomic library construction and sequencing

Total DNA was extracted and quantified as documented in the previous study^12^. All DNA samples were preserved at −80 °C until amplicon and metagenomic library preparation and sequencing. The detailed amplicon library preparation and sequencing have been documented previously^12,22^. Briefly, the V4-V5 regions of 16S rRNA genes were amplified using the universal primer set 515Y/926 R (5′-GTGYCAGCMGCCGCGGTAA-3′/5′-CCGYCAATTYMTTTRAGTTT-3′)^23^ with thermal cycling parameters followed the previously described protocol^23,24^. PCR products were used for library construction and subsequent sequencing on an Illumina NovaSeq platform at Novogene (Novogene, Beijing, China) using PE250 chemistries. For metagenomic sequencing, DNA was sheared into ~500 bp fragments using the Covaris Ultrasonicator M220 (Covaris, USA), then libraries were prepared using the NovaSeq Reagent Kit (Illumina, USA) according to the manufacturer’s instructions. Metagenomic sequencing was performed on the NovaSeq 6000 sequencing platform at Novogene (Beijing, China) using the Illumina PE150 chemistries.

### Sequence quality control

As previously described^12^, the raw reads of amplicon sequencing were first trimmed using cutadapt v3.5^25^ to remove adaptors and PCR primers with an error rate of 0.2, and the clean reads were subjected to further analysis using the Fuhrman lab pipeline^26,27^ with detailed parameters described previously by Huang *et al*.^12^. Briefly, clean reads were further split into 16S and 18 S rRNA pools using custom 16S/18 S databases derived from the SILVA 138 ribosomal RNA database^28^ and the Protist Ribosomal Reference database (PR^2^)^29^. The concatenated 16S rRNA reads were denoised using the DADA2^30^ denoise-paired command to reconstruct ASVs, which were then taxonomy classified against the SILVA v138 database^28^. ASV sequences of chloroplasts and mitochondria were removed in the following analysis. For Metagenomic sequencing, raw reads were first trimmed using fastp v0.19.5^31^, followed by the removal of human contaminants using bbmap.sh with specific parameters (minid = 0.95, maxindel = 3, bwr = 0.16, bw = 12, quickmatch, fast) and the recommended reference sequence file: hg19_main_mask_ribo_animal_allplant_allfungus.fa (http://sourceforge.net/projects/bbmap). Clean reads were used for metagenomic assembly and binning.

### Metagenomic assembly, gene prediction, MAG generation, refinement, and quality assessment

For each sample, high-quality reads were assembled into contigs using MEGAHIT v1.2.9^32,33^ with the kmer parameter–k-list 21,33,55,77,99,127. Samples from XS, DS and XB were also co-assembled using the same kmer set and assembler. The assembled contigs underwent gene-coding sequences prediction using Prodigal v2.6.3^34^ in “meta” mode. To generate a gene catalog of non-redundant sequences, all the coding sequences were clustered into representative sequences at 95% identity using CD-HT v4.6.1^35^. Functions of the non-redundant genes were predicted by KofamScan^36^ using the prokaryotic, eukaryotic and viral KEGG gene database (Release 106.1) with default settings.

Contigs longer than 1 kb were selected for metagenomic binning. We utilized multiple toolkits to recover high-quality MAGs, each sample assembly or co-assembly was binned using a combination of several tools including BASALT (via MetaBAT2 v2.12.1, MaxBin2 v.2.2.4, and CONCOCT v1.1.0 with more-sensitivity parameter)^37–40^, metaWRAP (via MetaBAT2 v2.12.1 and CONCOCT v1.1.0)^41^, MetaBinner v1.4.4^42^, MetaCoAG v1.1^43^, SemiBin v1.5.1 (single_easy_bin,–self-supervised)^44^, Vamb v4.1.0^45^ and MetaDecoder v1.0.18^46^ with default parameters. The resulting bins were then pre-assessed and quality-filtered using MDMcleaner v0.8.7^47^, retaining only bins with completeness ≥50% and contamination ≤10%. All these bins were further dereplicated into unique MAGs using dRep v3.4.0^48^ (-comp 50 -con 10 options) at 99% ANI. The completeness and contamination were estimated using CheckM v.1.2.1^49^, based on which these MAGs were classified into high-, medium-quality classes according to the MIMAG criteria^15^.

### Taxonomic annotation and phylogenomic analysis

The final 1887 MAGs were taxonomically classified using GTDB-Tk v2.1.1 with the reference GTDB release 214^16^. The archaeal and bacterial phylogenomic trees were constructed using protein sequences of 41 single-copy marker genes extracted from these MAGs^50,51^. Sequences were aligned using MAFFT v7.520^52^ and further automatically trimmed using trimAL v1.4.1 (-automated1)^53^. The alignments were concatenated using catfasta2phyml v1.1.0 (https://github.com/nylander/catfasta2phyml) and missing data were filled with gaps. The maximum-likelihood (ML) phylogenomic trees were constructed using IQ-TREE v2.0.3 with 1000 bootstrapping (-m LG + R10 -B 1000)^54^, and were visualized and annotated using the Interactive Tree of Life (iTOL) web tool^55^.

## Data Records

Raw reads generated in this study have been deposited at the NCBI Sequence Read Archive (SRA) database under the BioProject number PRJNA880762^56^, including accession numbers for both amplicon and metagenomic sequencing reads. MAGs have been deposited at Genbank under the same NCBI Bioproject^56^. ASVs, metagenomic assemblies and MAGs generated in this study have been deposited at Figshare^57^. The functional annotations of both contigs and MAGs have also been deposited into the same Figshare repository^57^.

## Technical Validation

All raw data processing steps, including software and parameters used in this study, were described in the Methods section. The quality of clean reads was assessed using FastQC v0.11.8, and the quality of the MAGs was assessed using CheckM v.1.2.1^49^. We have performed gene annotation of MAGs using Prokka v1.14.5^58^. MAGs recovered in this study were compared with diverse SCS habitats including cold seeps^17^, deep-sea sediments^18^, subtropical estuaries^19^, as well as OceanDNA^20^ and Tara Oceans^21^ using dRep v3.4.0^48^ (-comp 50 -con 10 options) at 95% average nucleotide identity to investigate the novelty of the MAGs.

### Supplementary information

Supplementary Information

Table S2

Table S3

Table S1

## Data Availability

All versions of third-party software and scripts used in this study are described and referenced accordingly in the Methods section.
