# Supplementary material for: Recovery of 1887 metagenome-assembled genomes from the South China Sea
Source: Sci Data. 2024 Feb 13;11:197. doi: 10.1038/s41597-024-03050-4 (PMC10864278; doi:10.1038/s41597-024-03050-4)
Supplement: Supplementary file 1 — Supplementary Information [file 41597_2024_3050_MOESM1_ESM.docx]

**Supplementary Information**

**Table S1.** Metadata of samples collected in the Xianbei seamount region, Dongsha, and Xisha areas.

**Table S2.** Relative abundance and taxonomy of 16S rRNA gene amplicon sequences recovered from this study.

**Table S3.** Comparison of MAGs recovered from this study with those from diverse marine environments.
